# Schule aus dem Geist von Public Health? Schulische Gesundheitsförderung aus bildungssoziologischer Perspektive

**DOI:** 10.1007/s00103-022-03547-6

**Published:** 2022-06-01

**Authors:** Uwe H. Bittlingmayer, Gözde Okcu

**Affiliations:** Institut für Soziologie, Pädagogische Hochschule Freiburg, Kunzenweg 21, 79117 Freiburg im Breisgau, Deutschland

**Keywords:** Gesundheitsförderung in der Schule, Bildungssoziologie, Inklusion, Gesundheitsungleichheiten, Bildungsungleichheit, Health promotion in schools, Sociology of education, Inclusion, Health inequality, Educational inequality

## Abstract

Im Rahmen der Gesundheitsförderung im Sinne der Ottawa-Charta wird der Schule eine Reihe von Aufgaben und Funktionen zugeschrieben, die unter anderem zur Reduktion von gesundheitlichen und sozialen Ungleichheiten beitragen sollen. Dabei finden allerdings in der Public-Health-Diskussion zur schulischen Gesundheitsförderung wichtige theoretische Überlegungen und empirische Erkenntnisse aus der Bildungssoziologie, die Schule als eine hierarchisierende, segregierende, Ungleichheiten produzierende und reproduzierende Institution analysiert, kaum Platz. In diesem Diskussionsbeitrag werden einige bildungssoziologische Positionen und die normative Rahmung der schulischen Gesundheitsförderung vorgestellt. Ferner wird auf die Widersprüche zwischen den Zielen der Gesundheitsförderung und den aktuellen schulischen Bedingungen eingegangen. Zum Schluss werden konzeptionelle Überlegungen für eine Perspektive vorgestellt, die Bildung aus dem Geist der Gesundheitsförderung entwickelt, dabei werden Verbindungen zu Inklusion, Demokratiebildung sowie Menschenrechten hergestellt.

## Abstract (en)

In the context of health promotion in terms of the Ottawa Charter, a series of tasks and functions are attributed to schools, including the reduction of health and social inequalities. However, important theoretical considerations and empirical findings from the sociology of education, which analyzes school as a hierarchizing, segregating, inequality-producing, and reproducing institution, hardly find place in the public health debate on school health promotion. In this discussion article, some positions on schools from the sociology of education and the normative framework for health promotion in schools are presented. Furthermore, the contradictions between the goals of health promotion and the current conditions in schools, are discussed. In conclusion, some conceptual considerations are introduced from a perspective that rethinks education from the health promotion point of view and connects with inclusion, democracy education, and human rights.

## Einleitung

Es gibt kaum einen Bereich innerhalb der Gesundheits- und Sozialwissenschaften, in dem die positive Bedeutung so einhellig herausgestellt wird, wie im Bereich der schulischen Gesundheitsförderung. Ausgehend von der Ottawa-Charta zur Gesundheitsförderung der Weltgesundheitsorganisation (WHO) lässt sich die Schule als besonders bedeutsames *Setting *deklarieren, das über die allgemeine Schulpflicht, zumindest in OECD-Ländern wie Deutschland, tatsächlich alle Kinder erreicht. Die Schule kann deshalb und wird im Normalfall als Interventionsort genutzt, um *Gesundheitsförderung im Setting *zu betreiben. Die praktische Umsetzung der Entwicklung eines gesundheitsförderlichen Settings Schule, in dem alle Prozesse daraufhin abgestimmt werden, dass sie zur Gesundheitsförderung aller systematisch im Setting anzutreffenden Personen unmittelbar und mittelbar beitragen (von den Schüler*innen über das pädagogische Personal bis hin zu den Eltern, Hausmeister*innen und dem Reinigungspersonal), erfolgt dabei in aller Regel selbst in der Public-Health^1^-Perspektive kaum [1].

Die deshalb von Beginn an normativ abgespeckte Variante der Gesundheitsförderung im Setting Schule (vgl. hierzu bereits [1]) erfährt, nachdem sie in Deutschland in den 1990er- und 2000er-Jahren einen ersten Höhepunkt verzeichnen konnte, international eine starke Renaissance, die seit einigen Jahren nach Deutschland zurückwirkt. Dabei ist der erneute Fokus auf die schulische Gesundheitsförderung eng mit der über nunmehr 2 Jahrzehnte andauernden Diskussion über Gesundheitskompetenzen (engl.: „health literacy“) verbunden [2, 3]. Schulische Gesundheitsförderung soll aktuell gewissermaßen automatisch eine Doppelfunktion übernehmen: die Steigerung der individuellen Gesundheits*ressourcen* einerseits sowie die Steigerung der individuellen Gesundheits*kompetenzen* andererseits. Die Institution und Organisation Schule tritt dabei vorrangig als Vermittlerin von individuell und gesellschaftlich wertzuschätzenden Kompetenzen auf. Die der Schule inhärenten Widersprüche und Ambivalenzen, die symbolische Gewalt pädagogischer Standardisierungen [4, 5] oder die durch die Institution Schule produzierten sozialstrukturellen Hierarchien [6] werden selten Thema.

An diesem Punkt möchte der vorliegende Diskussionsbeitrag ansetzen. Wir möchten in einem ersten Schritt einige theoretische Positionen aus der Bildungssoziologie und empirische Befunde aus der Bildungsforschung vorstellen, auf die in der Public-Health-Debatte um schulische Gesundheits(kompetenz)förderung noch immer selten zurückgegriffen wird. Wir werden dann in einem zweiten Schritt an die umfassenden normativen Rahmungen erinnern, mit denen schulische Gesundheitsförderung im Sinne der WHO verbunden wird. Anschließend werden wir einen Perspektivwechsel vollziehen und einige konzeptionelle Überlegungen vorstellen. Die Fragestellung lautet dann: Wie müsste Schule aussehen, wenn Gesundheitsförderung im Sinne der WHO ernst genommen wird? In einer kurzen Schlussbetrachtung werden die wichtigsten Argumente resümiert.

## Schule und schulische Gesundheitsförderung aus bildungssoziologischer Perspektive

Die systematische Förderung der Gesundheit von Kindern und Jugendlichen in der Schule trifft auf eine Institution, die außerhalb der schulischen Institutionen liegende *soziale Ungleichheiten* in den sozioökonomischen Ressourcen und der soziokulturellen Herkunft *reproduziert*. Solche Bildungsungleichheiten sind in der Bildungssoziologie seit den 1960er-Jahren präsent und kontinuierlich diskutiert worden (vgl. [7]), haben aber in Deutschland erst seit den international vergleichenden Studien wie PISA, TIMSS und IGLU wieder ein erstaunliches Ausmaß massenmedialer Aufmerksamkeit erreicht. Das deutsche Bildungssystem gilt dabei im internationalen Vergleich als ganz besonders selektiv – in kaum einem anderen OECD-Land spielt die soziale Herkunft für den Bildungserfolg, zumeist gemessen an der Schulformzugehörigkeit und/oder den schulischen Performanzen, eine so bedeutsame Rolle wie in Deutschland (vgl. u. a. [8]). Neben den in Deutschland besonders durchschlagenden Herkunftseffekten lassen sich weitere problematische Dimensionen des deutschen Bildungssystems bestimmen, *die einer schulischen Gesundheitsförderung von vornherein abträglich sind*.

So hält Deutschland in vielen Bundesländern nach wie vor fest an einem sozial segregierten, nach Ansicht der meisten Bildungssoziolog*innen diskriminierenden und die biografischen Chancen erheblich einschränkenden Förderschulsystem, das sich gegenüber der Absicht schulischer Inklusion erfolgreich hat zur Wehr setzen können (vgl. u. a. [9]). Die Prozesse, die sich im Rahmen der (deutlich überdurchschnittlichen) Überweisung von Migrant*innenkindern auf die Förderschule vollziehen, sind bereits vor 20 Jahren von Mechtild Gomolla und Frank-Olaf Radtke als „institutionelle Diskriminierung“ bezeichnet und beschrieben worden [10–12]. Auch die im internationalen Vergleich besonders frühe Trennung nach der 4. Schulklasse (in Berlin und Brandenburg ab der 6. Schulklasse) und Aufteilung in hierarchisch gegliederte Schulformen trägt dazu bei, dass schulische Gesundheitsförderung und schulische Gesundheitskompetenzförderung insbesondere in den statusniedrigen Schulzweigen von Beginn an unter Bedingungen sozialer Benachteiligung stattfinden und in dieser Hinsicht kompensatorisch agieren müssen (die Frage, inwieweit Schule überhaupt in der Lage ist, außerhalb ihrer selbst produzierte soziale Ungleichheitsverhältnisse zu kompensieren, hat Basil Bernstein [13] kritisch diskutiert).

Schließlich wird in der Bildungssoziologie darauf hingewiesen, dass – nicht nur in Deutschland – auch die Lehrkräfte einen eigenständigen Effekt bei der Produktion von Bildungsungleichheiten innehaben, insofern reproduziert Schule nicht nur außerhalb ihrer selbst liegende soziale Ungleichheiten, sondern sie produziert sie auch (vgl. z. B. [14]). Das gilt insbesondere für die Übergangsempfehlungen, bei denen bei sozial unterprivilegierten Schüler*innen außerhalb der von den Schüler*innen selbst erbrachten Leistungen und nachweisbaren schulischen Kompetenzen Kriterien wie fehlende spätere soziale/elterliche Unterstützung oder Deutsch als Zweitsprache auf dem Gymnasium hinzugezogen werden, um die Empfehlung für eine Schulform außerhalb des Gymnasiums zu begründen. Die in dieser Hinsicht bahnbrechenden IGLU-Studien konnten sehr genau zeigen, inwieweit die soziale Herkunft die für eine Gymnasialempfehlung notwendigen Kompetenzen in Deutsch und Mathematik beeinflusst (vgl. z. B. [15, 16]).^2^ Das gilt aber auch für die Bewertung des Schüler*innenverhaltens, das geschlechtsdifferenten Mustern folgt und etwa das häufig schulkonformere Verhalten von Mädchen positiv sanktioniert.

Bereits diese wenigen Hinweise auf Resultate der empirischen Bildungsforschung können verdeutlichen, dass die Absicht, die Gesundheit von Schüler*innen in der Schule nachhaltig zu fördern, auf eine institutionelle und strukturelle Rahmung trifft, die – vorsichtig formuliert – nicht auf die Optimierung der Gesundheitsressourcen programmiert ist. Über den empirischen Nachweis von bestehenden Bildungsungleichheiten hinaus liefert die Bildungssoziologie 3 übergreifende Problemhorizonte, die für schulische Gesundheitsförderung aus unserer Sicht unmittelbar relevant sind.

*Erstens *ist eine der wichtigsten, wenn nicht sogar die wichtigste Funktion der Schule, Kinder und Jugendliche mit Blick auf die in der Gesellschaft verfügbaren unterschiedlich privilegierten sozialen Positionen entlang ihrer schulischen Leistungen zu selektieren und in die bestehenden Hierarchien ohne Widerstand einzugliedern (vgl. etwa [17]). Pierre Bourdieu bezeichnet das als die Umstellung von der unmittelbar familialen Vererbung der verfügbaren Handlungsressourcen zum schulischen Reproduktionsmodus sozialer Klassenverhältnisse. Dieser schulische Reproduktionsmodus ist dabei weniger deterministisch als die direkte familiale Übertragung und gerade in Zeiten massiver Bildungsexpansion volatiler und unschärfer; das heißt, weder sind die unterprivilegierten Bevölkerungsgruppen schicksalhaft auf die unteren sozialen Positionen festgelegt, noch gelingt die Statusreproduktion aller Kinder der besonders privilegierten Gruppen bruchlos. Es gibt das, was die Bildungssoziologie die „unwahrscheinlichen Bildungsverläufe“ nennt [18].

Aber massiver Bildungsaufstieg und massiver Bildungsabstieg gegenüber der Elterngeneration sind nach wie vor die Ausnahme von der Regel. Die in Deutschland immer noch besonders ausgeprägten Bildungsungleichheiten und die unterschiedlich auf die Anforderungen der Schule vorbereiteten sozialen Milieus [19] treten aber durch den formalen Charakter schulischer Prozesse in den Hintergrund. Auf diese Weise wird eine auf der Ideologie der Meritokratie aufgebaute soziale Hierarchie mit einer enormen Legitimität versehen und insbesondere von den unterprivilegierten Gruppen selbst nicht infrage gestellt, weil sie ja in der Schule erfahren und gelernt haben, dass die anderen Kinder und Jugendlichen „es besser können“. Die innerhalb von kompetitiven Gesellschaften wie der deutschen notwendige Selektions- und die mit der Akzeptanz bestehender sozialer Hierarchien verbundene Legitimationsfunktion sind nach unserer Auffassung nicht im Horizont der Konzepte schulischer Gesundheitsförderung enthalten. Dabei ist einerseits zu beachten, dass die Kopplung der sozialen Verteilung von begehrenswerten Gütern (in der Regel durch Einkommensverteilungen ausgedrückt) an Bildungstitel sozial willkürlich und keineswegs zwingend ist, und andererseits, dass auch innerhalb des akademischen Feldes akademische Titel im Durschnitt mit ganz unterschiedlichen Privilegien verbunden sind (sichtbar im Vergleich eines Mastertitels in Kindheitspädagogik, Physik oder Jura).

*Zweitens *lässt sich die Schule nach Michel Foucault [20], motiviert durch eine Kritik an den Disziplinierungsformen, mit der eine Gesellschaft ihre Reproduktion (nicht intentional) sicherstellt, als eine durchdringende Technik der Macht beschreiben, der es um die Produktion einer bestimmten Form von Individuen geht (vgl. für eine kluge Übertragung des foucaultschen Ansatzes in Public Health [21]). Die Schule als Institution folgt strukturell der sozialen Logik des Militärischen, der es um die Zurichtung von disziplinierten Individuen geht, die im Zweifelsfall, ohne zu fragen, gehorchen und zum Beispiel die körperlichen Impulse unter Kontrolle bringen. Die Schule ist darüber hinaus eine Überwachungsanstalt, in der Praktiken der überwachenden Hierarchie, der normierenden Sanktion und der kontinuierlichen Prüfung dazu führen, dass aus Kindern Individuen und aus Individuen pädagogisch bearbeitbare Fälle werden. Schule ist damit Foucault zufolge weniger eine gesellschaftliche Institution, die sicherstellen soll, dass allen Kindern und Jugendlichen das Höchstmaß an qualitativ hochwertiger Bildung zuteilwird, sondern von Beginn an eine Disziplinarinstitution, die Menschen nach den Bedürfnissen existierender Gesellschaften „abrichtet“ (siehe auch [22, 23]). Dieser Disziplinierungsaspekt findet sich in den Konzepten schulischer Gesundheitsförderung ebenfalls nicht wieder.

*Drittens *schließlich wird aus bildungssoziologischer Perspektive selbst die positive Grundbedeutung von Bildung in den Gesundheitswissenschaften herausgefordert. In Public Health wird Bildung in aller Regel als eine für die individuelle Aufrechterhaltung von Gesundheit äußerst bedeutsame Handlungsressource konzeptionalisiert. Je mehr Bildung ein Subjekt akkumulieren kann, desto besser für seine Gesundheit. Das gipfelt in sozialepidemiologischen Berechnungen, die etwa Männer und Frauen mit Abitur eine um 3 bzw. 4 Jahre erhöhte Lebenserwartung attestieren [24]. Die deutschen Versicherer verweisen sogar darauf, dass Hochgebildete bis zu 12 Jahre länger leben als „Ungebildete“ [25]. Diese Befunde führen dann zu Überlegungen, dass soziale Ungleichheiten und damit verbundene gesundheitliche Ungleichheiten durch eine massive Bildungsexpansion bzw. einen gesellschaftsweiten Anstieg kulturellen Kapitals signifikant zu reduzieren wären [26, 27].

Diese Vorstellung ist trügerisch: Bereits Raimond Boudon [28] zeigt, dass die Expansion des Bildungssystems wenig mit einer sozial gleicheren Gesellschaft zu tun hat und Bildungsexpansion aus strukturellen Gründen nicht in der Lage ist, die vor allem in der Arbeitswelt hergestellten sozialen Ungleichheiten zu reduzieren. Michael Vester argumentiert auf dem Fundament der bourdieuschen Bildungs- und Ungleichheitssoziologie, dass es weiten Teilen der Bevölkerung mittlerweile gelungen ist, sich erfolgreich in Bildungsinstitutionen zu behaupten und ein signifikanter Anstieg der Hochschulzugangsberechtigten und Akademiker*innen in den Arbeitermilieus und im Kleinbürgertum zu verzeichnen ist. Allerdings ist dieser Anstieg des kulturellen Kapitals eben nicht mit einem sozialen Aufstieg zu verwechseln, sondern prallt an der Klassenstruktur der deutschen Gesellschaft ab [29, 30]. Das liegt zum einen an der relativen Entwertung von Bildungstiteln und zum anderen an den je nach sozialer Herkunft differenten Möglichkeiten, die erworbenen höheren Bildungstitel in lukrative Positionen zu transformieren.

Auch aus der Perspektive von Paul Willis [31] ist Bildung selbst keine unproblematische Zielgröße, sondern unmittelbar verbunden mit der Herrschaft sozialer Klassen. Dabei ist das Besondere an Willis’ berühmter Studie „Learning to labor“, dass sich hinter dem offensiven jugendlichen Protest gegenüber der Schule, der aus der üblichen Perspektive schulischer Gesundheitsförderung sicher als unvernünftig und gesundheitsabträglich bewertet würde, mit Blick auf die sicher eingeschränkten berufsbiografischen Chancen durchaus sinnvolle Strategien und Praktiken verbergen können, die auf ein wahrscheinliches körperlich anstrengendes Leben vorbereiten. Daraus folgt, dass sich selbst institutionell bildungsfeindliche Alltagsstrategien sozialer Milieus gerade auch aus Public-Health-Perspektive als sinnhafte Praktiken der Behauptung eigener Lebenswelten gegenüber nicht ernsthaft erreichbaren sozialen Normen und Zielgrößen verstehen lassen, Widerstand gegen Bildung also nicht automatisch als gesundheitsfeindlich abzuqualifizieren ist (das wird ausführlicher entwickelt in [4]).

Pierre Bourdieu schließlich zeigt, dass Bildungstitel, alltägliche hochkulturelle Bildungspraktiken und ein gebildeter Habitus, Bestandteile dessen, was er kulturelles Kapital nennt, eine eigenständige Form sozialer Herrschaft sind, bei denen die Schule soziale Klassenprivilegien umformatiert in individuelle Exzellenz ([5], vgl. z. B. [32, 33]). Die durch Bildung selbst vermittelte soziale Herrschaft führt zu einer Hierarchie des Akademischen gegenüber dem Nichtakademischen, die nicht in der Natur von Gesellschaften liegt, sondern die durch Prozesse der Verkennung privilegierter Startbedingungen – hier des Bildungsbürgertums und der ihm nacheifernden Mittelschichten – vermittelt ist. Die über Bildung vermittelte Herrschaft funktioniert als symbolische Herrschaft, das heißt, dass die unterprivilegierten sozialen Gruppen die soziale Welt und die ihr inhärenten Klassifizierungen aus der Perspektive derer betrachten, die sie dominieren und die Teilung in unterschiedlich wertvolle Fähigkeiten und Fertigkeiten durchsetzen (vgl. z. B. [34]). Nach Bourdieu besorgt gerade die Schule die Aufgabe der Verschleierung, sie verbirgt die Mechanismen der Macht. Die „schulischen Inszenierungen des Lernens und der Privilegierung sprachlicher Kommunikation sind Ausdruck einer sozialen Hierarchie geistiger und körperlicher Tätigkeiten. Deren Folge ist die massive Abwertung praktischer Fertigkeiten, konkreter Kenntnisse und einer korrespondierenden praktischen Intelligenz – und damit genau jener Lern- und Wissensformen, die in den kapitalschwachen Milieus vorherrschend sind“ [35].

Die hier nur sehr kursorisch vorgestellten übergreifenden herrschaftskritischen Perspektiven auf Schule und Bildung zeichnen gegenüber der Idee, dass Schule ein vorzüglicher Ort der Gesundheitsförderung ist oder zumindest sein könnte, ein anderes Bild. Um den Kontrast beider Sichtweisen auf dieselbe Institution zu schärfen, werden wir im nächsten Abschnitt kurz den normativen Horizont schulischer Gesundheitsförderung, wie ihn die WHO programmatisch bis heute formuliert, in Erinnerung bringen.

## Die normativen Rahmungen schulischer Gesundheitsförderung

Die normativen Ansprüche einer an die WHO angelehnten schulischen Gesundheitsförderung sind gewaltig und sehr umfangreich. Nicht allein bei der aktiven Einbindung *aller *relevanten Akteure in eine gesundheitsfördernde Schulentwicklung, sondern auch in Hinblick auf die Umsetzung eines Setting-orientierten Ansatzes. Seit der ersten Internationalen Konferenz zur Gesundheitsförderung in Ottawa 1986 wird der Settingansatz als Schlüsselstrategie in internationalen und nationalen Abkommen und Gesetzen zur Gesundheitsförderung aufgeführt. Beispiele hierfür sind die Jakarta-Deklaration von 1997, die Bangkok-Charta von 2005 oder auch das sogenannte Präventionsgesetz von 2016. Auch wenn der Settingansatz seit fast 40 Jahren, seit der Ottawa-Charta, politisch die Kernstrategie für die Gesundheitsförderung ist, sind Beispiele für eine vollständige Umsetzung des Settingansatzes im Setting Schule bis heute in Deutschland sehr selten. Als Indikator könnte hier dienen, dass in der Good-Practice-Datenbank des Kooperationsverbunds Gesundheitliche Chancengleichheit [36] der Anteil der schulischen Projekte, die das Kriterium Settingansatz erfüllen, äußerst dürftige 1,6 % beträgt (11 Projekte von insgesamt 696 Projekten im Setting Schule). Trotz der Betonung des Settingansatzes im politisch-rechtlichen Kontext überwiegen an Schulen kurzzeitige aufklärerische Gesundheitsförderungsmaßnahmen zu den Themen Ernährung, Bewegung, Sucht oder Zahngesundheit.

Darüber hinaus gibt es in der Regel aus der Suchtprävention stammende Gesundheitsförderungs- und Präventionsprogramme, die über einen längeren Zeitraum angelegt sind und die statt oder neben informationsvermittelnden Maßnahmen auf die Förderung von Lebenskompetenzen (engl.: „life skills“) abzielen. Diese Programme – wie „Lions-Quest – Erwachsen werden und Erwachsen handeln“, „Pausenlos gesund“ von der Stiftung Gesundheitswissen, das „Buddy-Projekt“ der Vodafone-Stiftung oder „ALF – Allgemeine Lebenskompetenzen und Fertigkeiten der Arbeitsgruppe Präventionsforschung“ am Institut für Therapieforschung (IFT), um nur einige wesentliche zu nennen – kommen zwar einer Setting-orientierten Perspektive am nächsten, indem sie die individuelle Kompetenzentwicklung adressieren und auf diese Weise die Kompetenzebene des Settingansatzes abdecken. Die *beiden weiteren Dimensionen des Settingansatzes* – die *gesundheitsförderliche Gestaltung der Lebenswelten* und *Partizipation* – werden in den meisten Programmen, wenn überhaupt, nur am Rande bearbeitet. Auch den Lehrpersonen ist die normative Reichweite des Settingansatzes wenig geläufig.

Wie weitreichend schulische Gesundheitsförderung von der WHO gedacht wird, erschließt ein Blick auf die 12 Kriterien, die die WHO für eine gesundheitsfördernde Schule festgelegt hat^3^ (Tab. 1). Ohne zu stark in Details gehen zu wollen, existiert nach dem besten Wissen der Verfasserin und des Verfassers kein Programm zur schulischen Gesundheitsförderung, das diese Liste vollumfänglich erfüllt. Auch die für den deutschen Sprachraum konzeptionell stärksten Ansätze von Peter Paulus und Kevin Dadaczynski können die strukturellen Widersprüche, in die eine schulische Gesundheitsförderung unter aktuellen Bedingungen der kompetitiven Beschulung gerät, nicht einfangen [40, 41]. Die hier formulierten Kriterien sind mit Blick auf die Lebenskompetenzen aller (!) Schüler*innen (Nr. 1, Nr. 2), die Offenlegung der selektierenden Funktionen von Schule (Nr. 3), die Betonung der Gesundheit und des Wohlbefindens (engl.: „well-being“; Nr. 8) oder die Einbeziehung des schulischen Umfelds im Sinne eines sozialökologischen Zugangs (Nr. 6, Nr. 7, Nr. 11) so formuliert, dass sie zu den im vorherigen Abschnitt vorgestellten Befunden und theoretischen Betrachtungen in einem ziemlich offensichtlichen Widerspruch stehen.

**Table 1:** 

1	Die aktive Förderung des Selbstwertgefühls der Schüler, indem deutlich gemacht wird, dass jeder Einzelne zur Gestaltung des Schulalltages beitragen kann
2	Die Entwicklung guter Beziehungen im Alltag der Schule, zwischen dem Schulpersonal und den Schülern und unter den Schülern selbst
3	Die Klärung des gesellschaftlichen Auftrags und der Ziele der Schule für das Schulpersonal und die Schüler
4	Die Bereitstellung einer Vielfalt von Aktionsmöglichkeiten zur Aktivierung aller Schüler
5	Die Nutzung jeder Gelegenheit zur Verbesserung der physischen Umwelt der Schule
6	Die Entwicklung guter Kontakte zwischen der Schule, dem Elternhaus und dem kommunalen Umfeld
7	Die Entwicklung guter Kontakte zwischen den örtlichen Grund- und weiterführenden Schulen zur Aufstellung eines kohärenten Lehrplanes zur Gesundheitserziehung
8	Die aktive Förderung der Gesundheit und des Wohlbefindens der Schüler und des Schulpersonals
9	Die Überprüfung der Rollen des Schulpersonals als gesundheitliche Vorbilder
10	Die Überlegung, inwieweit die Schulmahlzeiten (falls angeboten) auch zur Ergänzung des Lehrplanes zur Gesundheitsbildung und Gesundheitserziehung genutzt werden können
11	Die Nutzung der Angebote der kommunalen Dienste zur Beratung und Unterstützung der Gesundheitsbildung und Gesundheitserziehung
12	Die Weiterentwicklung der Schulgesundheitsdienste und deren Vorsorgeuntersuchungen zu einer aktiveren Unterstützung der Gesundheitsförderung im gesamten Lehrplan

Dieser Widerspruch wird insbesondere dann virulent, wenn es um den *gleichberechtigten Einbezug* der ressourcenschwachen Schüler*innen geht, derjenigen Schüler*innen, die in die unteren Formen der bildungsinstitutionellen Statushierarchien vorsortiert werden, die aufgrund mangelnder deutscher Sprachkenntnisse und einer monolingualen Ausrichtung des deutschen Bildungssystems benachteiligt sind, kurzum, wenn es um diejenigen geht, die im schulischen Konkurrenzkampf unterliegen (vgl. hierzu auch [42]). Wenn für diese Gruppen von Schüler*innen tatsächlich Gesundheit und Well-Being im Sinne der WHO, also ein Zustand körperlichen, geistigen, seelischen und sozialen Wohlbefindens, adressiert werden soll, dann ist das unter den gegebenen Rahmenbedingungen der Beschulung in Deutschland, dazu gehören etwa die systematische Ungleichheitsreproduktion und -produktion, Leistungsorientierung, kompetitive und disziplinierende Grundausrichtung, so unsere Überzeugung, nicht realisierbar. Im nächsten Abschnitt möchten wir die Analyseperspektive umdrehen und skizzieren, wie ein Schulsystem aussehen könnte, das zu den Forderungen und normativen Ansprüchen einer gesundheitsfördernden Schule besser kompatibel wäre.

## Schule aus dem Geist von Public Health

Wir haben uns seit etwa 3 Jahrzehnten zunehmend an den Gedanken gewöhnt, dass Bildung das wichtigste Gut sei, weil Individuen mithilfe von Bildung eine vernünftige und autonome Konzeption des guten Lebens entwickeln, eine halbwegs krisensichere Berufsbiografie erwarten und demokratische Teilhabe realisieren können. Individuelle Bildungsanstrengungen scheinen der Universalschlüssel zu sein für gesellschaftliche Partizipation. Allerdings sind Gesundheit oder demokratische Mitbestimmung, ganz unabhängig von schulischen Leistungen, Menschenrechte bzw. Grundrechte, die selbst dann Bestand haben müssen, wenn Menschen kognitive Einschränkungen aufweisen oder mit motivationalen Problemen zu kämpfen haben, sich in der Leistungsgesellschaft zu behaupten (vgl. z. B. [43]). Die Konzeption von individueller Bildung als Bedingung der Möglichkeit angemessener gesellschaftlicher Teilhabe scheitert daran, dass Menschenrechte oder Grundrechte eben nicht über Leistungskontexte definiert werden dürfen. Vor diesem Hintergrund ist es aus unserer Sicht reizvoll, den Bedingungszusammenhang zwischen Bildung und Gesundheit (natürlich gibt es deutlich mehr Dimensionen, die einzubeziehen wären, aber das blenden wir hier einmal aus) umzudrehen und Schule bzw. Bildung aus der Perspektive des individuellen Anspruchs auf Gesundheit, das heißt auf einen Zustand körperlichen, geistigen, seelischen und sozialen Wohlbefindens, zumindest anzudenken – im Sinne einer strukturellen schulischen Gesundheitsförderung.

Wie zahlreiche Studien aus den letzten Jahren belegen, steigt der Leistungsdruck bei Schüler*innen und ihren Eltern deutlich an und führt zu steigenden Zahlen psychischer Erkrankungen und anderen problematischen Outcomes [44, 45]. Vor diesem Hintergrund wären schulische Bildungsinstitutionen so zu konzipieren, dass Leistungsdruck gar nicht erst entsteht. Hier wäre an die Abschaffung von Ziffernnoten ebenso zu denken wie an die Abschaffung pädagogisch sinnloser Klassenwiederholungen, die durch gezielte Förderung in den schwächeren oder in der Konzentration auf die bereits guten Kompetenzen der Schüler*innen ersetzt werden müssen. Dass Schule ganz ohne Ziffernnoten gut funktioniert, zeigen „Versuchsschulen“ wie etwa die Bielefelder Laborschule – bislang hat sich nur noch niemand getraut, diese Erkenntnis flächendeckend umzusetzen.

Für Deutschland gilt aus Public-Health-Perspektive, dass die Aufrechterhaltung einer starren Schulhierarchie, die ständischen Mechanismen folgt [30], nicht zu legitimieren ist und durch eine Schule für alle zu ersetzen wäre. Zu überlegen wäre auch, inwieweit das System der Zwangsindividualisierung durch permanente Leistungsüberprüfungen [20] insgesamt abzuschaffen wäre, weil – zumindest in den hoch innovativen gesellschaftlichen Feldern – Kooperation als Schlüssel zu Innovationen gilt.

Inhaltlich und schuldidaktisch wäre die noch immer schultragende Aufteilung in unterschiedliche „Fächer“ zu überdenken, die die Schüler*innen dann in späteren Phasen der Beschulung selbstständig synthetisieren sollen – eine Kunst, die die wenigsten Lehrpersonen oder Hochschuldozierenden zustande bringen. Stattdessen wäre an den spontanen Interessen der Kinder, ihrer Neugierde und offenen Weltaneignung anzusetzen. Dass auch jungen Kindern nicht durch die standardmäßige Art der Beschulung die Autonomiespielräume zugestanden werden, die sie bereits ausfüllen könnten, zeigt die neuere Kindheitsforschung sehr deutlich (vgl. [46–48]).

Eine wichtige, oben bereits genannte Dimension des Settingansatzes ist Partizipation. Schule wäre aus Public-Health-Perspektive insgesamt massiv zu demokratisieren. Hier sind Ansätze der Demokratiepädagogik zielführend, die die Demokratisierung der Schule in einer kaum noch überschaubaren Anzahl von Veröffentlichungen proklamiert. Das Potenzial demokratiepädagogischer Konzepte wird in außercurricularen Unterrichtsprogrammen zur Life-Skills-Förderung durchaus systematisch aufgegriffen. Allerdings bleibt den Schulen überlassen, ob sie solche Programme nutzen oder nicht. Das Verhältnis zwischen Demokratie und Gesundheit ist insgesamt eines, das aus Public-Health-Perspektive aus unserer Sicht bislang stark vernachlässigt wurde (vgl. hierzu aber das posthum veröffentlichte letzte Buch von [49]). Das alleinige Eintreten für Demokratie und eine demokratisierte Schule reicht aus Public-Health-Perspektive so lange nicht aus, wie nicht gleichzeitig menschenrechtliche Perspektiven parallel thematisiert werden, weil sonst Mehrheitsentscheidungen im Setting Schule nicht mit gesundheitsfördernden Motiven der Minderheiten einhergehen müssen. Insgesamt müsste Schule als institutionalisierte Balance von Demokratie und Menschenrechten konzeptionalisiert werden (vgl. u. v. a. [50]).

Solche normativen Maximalforderungen, wie sie hier nur angedeutet werden können, rufen natürlich schnell Widerstand und Bedenken gegenüber der Realisierbarkeit, dem fehlenden Konsens der beteiligten Akteure, der fehlenden Finanzierung usw. auf den Plan. Es ist aber bemerkenswert, dass auch in der Erziehungswissenschaft und Soziologie mit dem Diskurs um Inklusion sehr ähnliche Zieldimensionen formuliert worden sind, die sich um die maximal mögliche Teilhabe von Kindern und Jugendlichen mit Einschränkungen drehen (einer der wichtigsten Autoren ist Georg Feuser; vgl. z. B. [9]). Allerdings gibt es bislang keine systematischen Bezugnahmen zwischen Public Health, (schulischer) Gesundheitsförderung und Inklusion. Gerade aus Sicht einer radikalen Perspektive auf schulische Gesundheitsförderung wäre die Wahrnehmung des Inklusionsdiskurses aus unserer Sicht äußert fruchtbar [51].

## Fazit

Innerhalb der schulischen Gesundheitsförderung wird die Institution Schule in der Regel als neutraler Interventionsraum konzeptionalisiert. Vergleichsweise selten werden die Widersprüche zwischen gerade dem deutschen Schulsystem und einer Perspektive, die konsequent auf schulische Gesundheitsförderung abzielt, offengelegt. In diesem Beitrag ging es zunächst darum, diesen Widerspruch mithilfe von bildungssoziologischen Befunden und Theorien zu verdeutlichen, um dann einen Perspektivwechsel anzuregen, der umgekehrt Bildungsinstitutionen aus dem Geist der Gesundheitsförderung betrachtet.

Diese hier nur angedeuteten Überlegungen zu einer Bildung, die auf dem Fundament der Gesundheitsförderung aufruht, wäre systematisch zu entfalten. Sie hätte das Potenzial, im Sinne Aaron Antonovskys, eine „upstream-policy“, also eine Politik, die an den Ursachen ansetzt und nicht an den Symptomen, zu bilden (vgl. [52]), die in der Lage wäre, nicht nachträglich an den Symptomen von durch Leistungsorientierung und Selektion verursachten Pathologien kurativ zu arbeiten, sondern Bildungsprozesse zu initiieren, die von vorneherein kompatibel mit dem Gesundheitsverständnis der WHO wären.

## Footnotes

1 Mit dem Begriff Public Health beziehen wir uns auf wissenschaftliche und politische Ansätze und Diskussionen um das Thema Gesundheit der Bevölkerung, insbesondere auf Gesundheitsförderung und Prävention im Sinne der Ottawa-Charta.

2 Dabei wäre es natürlich ein grobes Missverständnis anzunehmen, dass die Aufhebung der verpflichtenden lehrerseitigen Übergangsempfehlung von der Grundschule in die Schulformen der Sekundarstufe 1 und der Rekurs auf den Elternwillen zu einer Reduktion von Bildungsungleichheiten führt. Dieselben IGLU-Studien von Wilfried Bos et al. zeigen im Gegenteil, dass der Elternwille noch deutlich selektiver ist als die Empfehlung der Lehrkräfte.

3 Der hier zitierte Kriterienkatalog liefert aus unserer Sicht nach wie vor den präzisesten Überblick über die normative Reichweite umfassend verstandener schulischer Gesundheitsförderung. Aktuelle Kataloge und Modelle schulischer Gesundheitsförderung sind inhaltlich weitgehend deckungsgleich, verknüpfen die Kriterien aber mit Managementkonzepten oder Governance-Ansätzen. Vgl. hierzu etwa Literatur [37–39].
